# How are Brazilian university students coping with the COVID-19 pandemic? Results of an online survey on psychosocial well-being, perceived burdens, and attitudes toward social distancing and vaccination

**DOI:** 10.1371/journal.pone.0284190

**Published:** 2023-04-26

**Authors:** Aneliana da Silva Prado, Elisabeth Kohls, Sabrina Baldofski, Alessandra Sant’Anna Bianchi, Luciano Imar Palheta Trindade, Joanneliese de Lucas Freitas, Christine Rummel-Kluge

**Affiliations:** 1 Faculty of Life Sciences, Wilhelm Wundt Institute for Psychology, Leipzig University, Leipzig, Sachsen, Germany; 2 Department of Psychology, Federal University of Parana, Curitiba, Parana, Brazil; 3 Campus Curitiba, Federal Institute of Education, Science and Technology of Parana, Curitiba, Parana, Brazil; 4 Department of Psychiatry and Psychotherapy, Medical Faculty, Leipzig University, Leipzig, Sachsen, Germany; 5 Department of Psychiatry and Psychotherapy, University of Leipzig Medical Center, Leipzig, Sachsen, Germany; University of São Paulo, BRAZIL

## Abstract

**Background:**

The COVID-19 pandemic caused significant disruption to education systems worldwide, increasing pre-existing concerns regarding university students’ mental health. Brazil was among the countries most affected by COVID-19 cases and deaths and was considered a pandemic epicenter. This study aimed to investigate Brazilian university students’ mental health status and perceived burdens during the COVID-19 pandemic.

**Material and methods:**

From November 2021 to March 2022, a cross-sectional and anonymous online survey was conducted among students of a Brazilian federal university. Mental health status (depressive symptoms, alcohol and drug consumption) and social and emotional aspects in the pandemic context (social support, perceived stress, loneliness, resilience, and self-efficacy) were assessed with standardized measures. Students’ attitudes toward the COVID-19 pandemic and vaccination and perceived burdens of the pandemic were also investigated.

**Results:**

A total of *N* = 2,437 students completed the online survey. The PHQ-9 mean sum score was 12.85 (*SD* = 7.40), while *n* = 1,488 (61.10%) participants reported a sum score of 10 or more, indicating clinically relevant depressive symptoms. Further, *n* = 808 (33.1%) of the total sample reported suicidal thoughts. Levels of depressive symptoms, perceived stress, and loneliness were higher among undergraduate/bachelor students than doctoral students. Almost all participants (97.3%) reported being fully vaccinated against COVID-19. Multiple regression analyses showed that being single, having an income decreased during the pandemic, having a previous mental illness, having a chronic somatic condition, not finding positive aspects in the pandemic, lower self-efficacy, lower social support, lower resilience, and higher experienced loneliness were significantly associated with higher levels of depression.

**Conclusions:**

The study showed high levels of depressive symptoms and suicidal ideation among Federal University of Parana students. Therefore, health care providers and universities need to recognize and address mental health issues; psychosocial policies must be enhanced to mitigate the impact of the pandemic on students’ mental health and wellbeing.

## Introduction

The COVID-19 pandemic caused the most significant disruption to education systems in history, affecting an estimated 1.6 billion students in more than 190 countries [1]. Due to the pandemic, students had their face-to-face academic activities interrupted or changed to the online format and experienced an interruption of their social life and support. The closure of schools and other educational spaces affected about 94% of students worldwide and 99% of students in low- and middle-income countries [1]. Fear of a COVID-19 infection, the prevention measures, and overload of media information related to it, economic changes, interruption of academic activities, difficulties in adapting to online classes, uncertainty regarding academic development and success and professional future, and decrease in social interaction and support were some burdens pointed by the students [2–10].

In addition to health issues, the outbreak of the COVID-19 virus as a public health problem in 2019 and as a global pandemic from March 2020 onwards has also impacted people’s mental health around the globe. Collateral effects of social distancing and its outcomes have been broadly stated [11–20], and negative impacts such as feelings of despair, anxiety, confusion, anger, irritability, fear, frustration, and boredom have been reported among the general population [11–13, 20–24]. Besides, increased perceived stress, sleep disorders, post-traumatic stress symptoms, and suicidal ideation have also been described as possible outcomes of the pandemic [11, 13, 21, 23, 25–27], and less perceived support has been associated with suicidal ideation [23].

Regarding university students, some of the consequences of the pandemic include an increased socioeconomic and emotional vulnerability and a significant decrease in mental health, interpersonal relationships, and available social support [8, 28–30]. Before the pandemic, several studies pointed out a high prevalence of mental disorders in university students, drawing attention to the vulnerability of this population to emotional struggles [31–36]. Academic pressure, financial problems, lack of social support, and fear of the future are examples of some stressors present in student life [35–38]. Thus, student mental health concerns have increased since the pandemic outbreak.

In Brazil, the latest national survey about socioeconomic and cultural profiles of undergraduate students of federal institutions showed an increasing rate of undergraduate students’ emotional issues that influenced their academic performance. Anxiety, discouragement and demotivation, sleep disorders, helplessness or feelings of despair, loneliness, persistent sadness, and increased suicidal ideation over the years were reported [38].

Brazil was one of the countries most affected by COVID-19 cases and deaths. Until May 2022, the country had registered more than 30,564,536 cases and around 664,139 deaths (data available until May 8^th^, 2022; [39]). Social distancing was one of the measures adopted by the Brazilian government to flatten the infection rate curve [40, 41]. Despite the late start of vaccination in the country [42, 43] and the conflicts between the national government and state governments on the topic, due to a structured universal healthcare system and its immunization strategy, Brazil rapidly increased the vaccination status of its population as soon as the COVID-19 vaccine was available [44, 45]. Also, differing from other countries (e.g., India [46] and Iran [47] where vaccination hesitancy was high even among healthcare professionals), COVID-19 vaccine hesitancy was low among Brazilians [48].

As reported by the United Nations Educational, Scientific and Cultural Organization [1], the whole educational system was affected by social distancing measures worldwide, and there was no difference in Brazil [40, 41]. Brazil did not have lockdown measures comparable to European countries; nevertheless, most public universities and federal institutes stopped face-to-face activities in March 2020 and shifted to online teaching [40, 41, 49, 50].

In this context, an online cross-sectional survey in a Brazilian federal university reported difficulties related to time management and procrastination, studying, and performing physical activities as the main difficulties faced by undergraduate and graduate students during the pandemic [3]. Among graduate students, 51% addressed difficulties writing their master/doctorate thesis. Lack of motivation, lack of concentration, difficulties in writing and studying at home, anxiety in the face of the pandemic, and discouragement were the major perceived burdens regarding academic life [3].

An online cross-sectional survey revealed that Brazilian undergraduate students presented symptoms of depression (60.5%), anxiety (52.5%), and stress (57.5%) [51]. In a study of medical students from both public and private universities in Brazil, losses in concentration levels (79.5%) and academic performance (59.8%) were reported during the pandemic [9].

This study aims to provide a comprehensive overview of how the pandemic affected university students in Brazil in terms of their mental health status and perceived burdens.

The study design was based on an anonymous online survey conducted in Germany in 2020 [8] and 2021 [10]. Screening instruments for depressive symptoms, alcohol, and drug consumption were included. Social support, loneliness, self-efficacy, perceived stress, and resilience were included in the explorative study. Based on literature [5, 8, 10, 51], we hypothesize that students struggling with the burdens of the pandemic would, therefore, present significant sum scores for depressive symptoms, especially those with lower income. We also hypothesized that there would be differences in depressive symptoms and socioemotional aspects (loneliness, perceived stress, social support, self-efficacy, and resilience) between the COVID-19-related income changes (decreased income and increased income) and the four levels of courses that students were enrolled (undergraduate/bachelor courses, specialization/MBA/medical residency, master, and doctor courses).

## Material and methods

### Pandemic context of the study

The Federal University of Parana (UFPR), Brazil, has around 39,000 students and campuses in six cities in the state. It is the oldest Brazilian university, founded in 1912, and it has positioned itself as one of the most prestigious universities in the country [52]. It can be considered representative of public universities in Brazil and of the Federal Institutes regarding undergraduate/bachelor and graduate students.

When the survey took place, the COVID-19 situation was the following: on November 3^rd^, 2021, 54.68% of the population was fully vaccinated, the reproduction rate (R) of COVID-19 cases was 0.96, and the rate of new deaths per million was 1.05. When the survey ended on March 21^st^, 2022, 74.35% of the population was fully vaccinated, the reproduction rate (R) of COVID-19 cases was 0.82, and the rate of new deaths per million was 1.36 [53]. In Brazil, the Ministry of Education allowed resuming face-to-face academic activities on August 5^th^, 2021, under the observation of local regulations of states and municipalities–the higher education system had an exception and could not resume face-to-face teaching at that moment [54]. The UFPR was the first Brazilian federal university to resume face-to-face teaching on February 14^th^, 2022 [55]. Therefore, at the time of the survey, the sanitary measures to prevent contagion were milder, and face-to-face activities were taking place at the university again.

### Participants and procedures

The cross-sectional study was conducted online from November 3^rd^, 2021, to March 21^st^, 2022. The survey was set up in the online tool EFS Survey Unipark (Version 21.1) in Portuguese. All students at the university were invited via email, social media channels, and the university’s website to participate. The inclusion criteria were being currently enrolled as a student and 18 years or older. A total of *N* = 2,437 students completed the online survey. Undergraduate and graduate students from 12 sectors and five campuses at the Federal University of Parana participated in the study. The Ethics Committee of the UFPR approved the study (Register CAAE: 42886821.9.0000.0102, Approval no. 4.625.252). All participants provided an online informed consent via an opt-in function before participating, where they were informed about the voluntary nature of participation and the guarantee of anonymity.

### Measures

Questions related to sociodemographic and academic information (level of course, faculty, income, and change of income, residential situation, relationship status, migration background, and being a parent), chronic somatic conditions, times of personal and indirect social contact, as well as media and social media use, and media used for information about COVID-19 were asked. The four levels of courses were undergraduate/bachelor, specialization/MBA/medical residency (*lato sensu*), master, and doctor (both *stricto sensu*) courses. *Lato sensu* courses are graduate courses of short duration which are focused on professional development. *Stricto sensu* courses (master and doctor) are focused on research training. Moreover, the following measures were used:

### Mental health measures

The Patient Health Questionnaire-9 (PHQ-9; [56, 57]) was used to assess depressive symptoms over the past 14 days. The questionnaire comprises nine items on a 4-point Likert scale from 0 = "not at all" to 3 = "nearly every day," with a sum score ranging from 0 to 27, with higher scores indicating higher levels of symptoms (e.g., “Over the last two weeks, how often have you been bothered by any of the following problems? Little interest or pleasure in doing things?). A sum score of 10 or more indicated clinically relevant depressive symptoms. The item 9 (“thoughts that you would be better off dead, or of hurting yourself”) indicates suicidality when answered with a score of ≥ 1: "0 = Not at all," 1 = "Several days," 2 = "More than half the days," and 3 = "Nearly every day." The internal consistency of the PHQ-9 scale was excellent in the current sample: α = 0.90.

The hazardous alcohol use subscale of the Alcohol Use Disorders Identification Test (AUDIT-C; [58]) was used to assess alcohol consumption. On a 5-point Likert scale ranging from 0 = "never" to 4 = "4 or more times a week," the frequency of participants having alcoholic drinks was assessed (e.g., “How often do you have a drink containing alcohol?”–never, monthly or less, two to four times a month, two to three times per week, four or more times a week), the typical quantity they drink when consuming alcohol (0 = “1 or 2” up to 4 = “10 or more”), and the frequency of heavy alcoholic drinks consumed (i.e., six or more drinks on one occasion; 0 = "never" to 4 = "one a week”). The AUDIT-C total score ranges from 0 to 12, with higher scores indicating higher alcohol consumption and related risk. The internal consistency of the scale was acceptable in the current sample: α = 0.81. Besides, changes in drinking behavior during the pandemic were assessed (no change, drinking less, drinking more). To assess drug consumption, one AUDIT item was rephrased to "drug or substance abuse," and the potential change in drug consumption (no change, consuming less, consuming more) was also assessed. Besides, lifetime diagnosed mental disorders and current treatment of these disorders were examined. Further, the treatment for mental disorders during the months when the pandemic situation was more severe (in terms of mortality rates) in Brazil (from March to June 2021) was investigated.

Additionally, changes in body weight during the pandemic and their relation to the pandemic from the participant’s perspective were assessed.

### Social and emotional aspects of the COVID-19 pandemic

The ENRICHED Social Support Inventory (ESSI; [59]) was used to assess social support. It was translated to the Portuguese language using a back translation method. It has five items (e.g., “Is there someone available to whom you can count on to listen to you when you need to talk?”) rated on a 5-point Likert scale from 0 = “none of the time” to 4 = “all of the time,” with a total score ranging from 5 to 25, in which higher scores indicate a higher level of social support. In the current sample, the scale had excellent internal consistency, showing a Cronbach’s alpha coefficient of 0.93.

The UCLA 3-Item Loneliness Scale was used to assess experienced loneliness (e.g., “How often do you feel that you lack companionship?”) [60, 61]. The 4-point Likert scale ranges from 0 = “never” to 3 = “often” in each item, with a total score ranging from 0 to 9. Higher scores indicate more loneliness experienced. The internal consistency of the scale was good in the current sample: α = 0.86.

The perceived self-efficacy was assessed using the ten items General Self-Efficacy Scale (GSE; [62, 63]), which evaluates adaptation after experiencing stressful life events (e.g., “I can always manage to solve difficult problems if I try hard enough.”). It has a 4-point Likert scale ranging from 1 = “not at all true” to 4 = “exactly true,” with a composite score ranging from 10 to 40. Higher values indicate higher levels of self-efficacy. The internal consistency of the scale was excellent in the current sample: α = 0.90.

The Brief Resilience Scale (BRS; [64, 65]) was used to measure the ability to bounce back or adapt well in the face of adversity (e.g., “I tend to bounce back quickly after hard times.”), with a 5-point Likert scale ranging from 1 = “strongly disagree” to 5 = “strongly agree,”. The total score was ranging from 1 to 5. Higher values indicate higher levels of resilience. The internal consistency of the scale was good in the current sample: α = 0.86.

The Perceived Stress Scale (PSS-4; [66, 67]) was used to assess the degree to which situations in the individual’s life are appraised as stressful (e.g., “In the last month how often have you felt you were unable to control the important things in your life?”). The four items were answered on a 5-point Likert scale from 0 = “never” to 4 = “very often,” with a total score ranging from 0 to 16. Higher scores indicate more perceived stress. The internal consistency of the scale was acceptable in the current sample: α = 0.75.

The frequency of personal or indirect contact was assessed retrospectively regarding the period from March to June 2021, when the rates of new deaths per million reached their highest point in Brazil (14.54 on 01 April 2021, and 14.54 on 11 April 2021) [53]. The German research team developed the items [8, 10] and were translated to Portuguese using a back translation method and adapted to the Brazilian context. Some examples of the items are: “How many times per week did you personally meet with people (family members, friends, neighbors, etc.) beside your own household during the highest infection rate level (as from March to June 2021)?” and “How many times per week did you have indirect contact, e. g., via phone, with other persons (family members, friends, neighbors, etc.) beside your own household?”. These items were rated on a 6-point Likert scale from 1 = “not at all” to 6 = “multiple times a day.”

Furthermore, lifestyle aspects (social and cultural activity, healthy eating, dating behavior, and sexual activity) during the pandemic were assessed through the question: “We would like to ask you a few questions in what way the corona pandemic relates to your lifestyle. How do you evaluate your personally experienced restrictions in the following areas?–social and cultural activity, healthy eating, dating behavior, and sexual activity”. The items were translated to Portuguese using a back translation method.

### COVID-19 pandemic: Attitudes towards social distancing and vaccination, and perceived burdens

Questions regarding attitudes toward the COVID-19 pandemic and vaccination, stockpile behavior, psychosocial consequences, and perceived burdens as a result of the social distancing measures were asked. The items used in the German survey [8] were translated to Portuguese using a back translation method and then adapted to the Brazilian context (e.g., “I am worried because of COVID-19.”; “I am particularly at risk from the coronavirus due to existing medical conditions.”; “The pandemic is part of a larger conspiracy.”). The questions on attitudes were designed as single items reflecting single attitudes toward the pandemic and the related restrictions, therefore, no total score was computed.

### Statistical analysis

All analyses were conducted using IBM SPSS Statistics version 27.0. The level of significance applied to statistical testing was α = 0.05 (two-tailed). First, differences in sociodemographic variables were analysed between drop-outs with available sociodemographic data and completers, namely those participants who completed the survey until the last question of the Patient Health Questionnaire (PHQ-9). A one-way ANOVA was performed for continuous variable (age), and chi-squares analyses were performed for categorical variables (gender, being parent, migration background, relationship status, higher degree finished, level of currently enrolled course, change of income, income before the pandemic, and income after the pandemic). Second, descriptive statistics were performed for sociodemographic and socioeconomic variables. Students who did not report their income were only considered in descriptive analyses of the sample and when comparing completers’ and drop-outs’ sociodemographic characteristics. In this paper, the income is presented in Real (the Brazilian currency), which symbol is “R$.” At the moment this paper was written, 1 US$ (one dollar) was equivalent to R$ 5,18 (five reals and eighteen cents).

Third, one-way ANOVAs were performed to analyse potential differences in depressive symptoms (PHQ-9 sum score) between three groups of students with different self-rated COVID-19-related income changes (no change, decreased income, and increased income) and also between the four groups of students with different income ranges both before and after the pandemic (in the latter analyses, the students with no answer were not included because the goal was to evaluate the differences between all students who chose to report their income).

Further, a one-way ANOVA was performed to test for potential differences between students enrolled in four different levels of study (undergraduate/bachelor courses, specialization/MBA/medical residency, master, and doctor courses) in depressive symptoms (PHQ-9 sum score). A chi-squares analysis was performed to test if the number of students answering the PHQ-9 item 9 ≥ 1 (suicidality) differed between the four groups formerly mentioned. Bonferroni correction was applied to correct for multiple testing when applicable.

Following, one-way ANOVAs were performed to test for potential differences between students enrolled in four different levels of study (undergraduate/bachelor courses, specialization/MBA/medical residency, master, and doctor courses) in perceived stress (PSS-4 sum score), social support (ESSI sum score), loneliness (UCLA-3 sum score), self-efficacy (GSE sum score) and resilience (BRS sum score). All effect sizes were interpreted as suggested by Cohen [68].

A multiple linear regression analysis was performed to predict depressive symptoms (PHQ-9 sum score) based on the following predictors: sociodemographic variables (age, gender, relationship status, residential status, being a parent, migration background, change in income), mental health outcomes (loneliness–UCLA-3, social support–ESSI, self-efficacy–GSE, and resilience–BRS), lifetime mental disorder, chronic somatic condition, and social and emotional aspects (opinion about positive and negative aspects of the pandemic). Categorical variables with more than two categories were either dichotomized (relationship status, being a parent) or recoded into dummy variables (gender, migration background, change in income, lifetime mental disorder); gender was recoded into two new dummy variables: female and diverse because they presented higher mean scores for depressive symptoms (PHQ-9) than male participants, similar to previous studies. All predictors were entered simultaneously (enter-method). The assumption of no or little multicollinearity was not violated (Variance Inflation Factor [VIF] < 10; correlation matrix check, *r* ≤ .85) for multiple linear regression analyses. Perceived stress (PSS-4) was not included among the predictors due to missing data. The residuals for this regression model do not have a constant variance (heteroscedasticity) based on the scatterplot of the standardized residual and the standardized predicted value.

Finally, descriptive statistics were also performed for attitudes toward social distancing and vaccination, perceived burdens of the COVID-19 pandemic, mental health measures, and lifestyle and social and emotional aspects of the pandemic.

## Results

A total of *N* = 2,442 students completed the survey until the Patient Health Questionnaire (PHQ-9), covering the main study outcome measures. Of these, *n* = 5 were excluded for implausible data; therefore, the final sample comprised *N* = 2,437 participants. Sociodemographic information on the final sample is displayed in Table 1. Next to these completers, *n* = 807 were considered drop-outs. Sociodemographic data were available for *n* = 645 of the drop-outs.

**Table 1:** Sociodemographic characteristics of the total sample (*N* = 2,437).

Variable	*n*, %
Total	2,437 (100)
Gender	
Female	1,579 (64.8)
Male	821 (33.7)
Diverse	37 (1.5)
Age	
< 20 years	260 (10.7)
20–25 years	928 (38.1)
26–30 years	569 (23.3)
> 30 years	680 (27.9)
Relationship status	
In a relationship	1,349 (55.4)
Single	1,088 (44.6)
Residential status	
Alone	319 (13.1)
Not alone	2,118 (86.9)
Being parent	
Yes	335 (13.7)
No	2,102 (86.3)
Migration background	
Self	28 (1.2)
Parents	27 (1.1)
None	2,382 (97.7)
Income	
*Before COVID-19 pandemic*	
No income^a^	527 (21.6)
0–1100 R$/mo	549 (22.5)
1101–3300 R$/mo	796 (32.7)
> 3301 R$/mo	487 (19.6)
No answer	87 (3.6)
*After COVID-19 pandemic*	
No income^a^	380 (16.1)
0–1100 R$/mo	564 (23.1)
1101–3300 R$/mo	914 (37.5)
> 3301 R$/mo	507 (20.8)
No answer	72 (3.0)
Change in income	
Decrease	682 (28.0)
No change	1,354 (55.5)
Increase	401 (16.5)

^a^ Participants indicated not receiving any own income on a regular basis.

There were significant differences between drop-outs and completers in the following sociodemographic variables: age (completers were older), Welch’s *F*(1,1171.618) = 42.932, *p* < .001, η^2^ = 0.01; being parent, χ^2^(1) = 4.606, *p* = .032; level of currently enrolled course, χ^2^(3) = 23.24, *p* < .001; income before the pandemic, χ^2^(4) = 17.582, *p* = .001; and current income, χ^2^(4) = 26.159, *p* < .001. There were significant differences between drop-outs and completers regarding socioeconomic data: being a parent (10.5% in drop-outs, 13.7% in completers); being enrolled in undergraduate/bachelor (66.7% in drop-outs, 57.6% in completers) or in a doctor course (13.0% in drop-outs, 20.7% in completers); having an income range before the pandemic of R$ 0–1100 (27.8% in drop-outs, 22.5% in completers) or higher than R$ 3301 (14.4% in drop-outs, 19.6% in completers). After the pandemic, there were significant differences between having an income range of R$ 0–1100 (28.5% in drop-outs, 23.1% in completers), higher than R$ 3301 (14.4% in drop-outs, 20.8% in completers), and among those who did not want to report their income range (5.4% in drop-outs, 3.0% in completers).

Of the *N* = 2,437 students who completed the survey, *n* = 1,579 (64.8%) were female, *n* = 821 (33.7%) were male, and *n* = 37 (1.5%) diverse. The participants age ranged from 18–71 years old (*M* = 27.84, *SD* = 8.40); 97.7% (*n* = 2,382) declared not having a migration background; and 86.9% (*n* = 2,118) of the participants lived together with others.

Regarding their educational situation, *n* = 1,404 (57.6%) participants were currently enrolled in undergraduate/bachelor courses, *n* = 506 (20.8%) in master degree, *n* = 504 (20.7%) in doctor degree, and *n* = 23 (0.9%) in specialization/MBA/medical residency courses (*lato sensu* courses).

In total, *n* = 383 (15.7%) reported that they suffered from a chronic somatic condition, while *n* = 502 (20.6%) agreed or fully agreed with the statement that they were particularly at risk of coronavirus due to existing medical conditions.

The most common sources of income were by family support (*n* = 633, 20.8%), full-time job (*n* = 590, 19.4%), having a scholarship (*n* = 542, 17.8%), and having a paid internship (*n* = 233, 7.7%; multiple answers possible). Regarding the financial situation, *n* = 1,354 (55.5%) of the students reported that their income did not change during the COVID-19 pandemic, while *n* = 682 (28.0%) indicated a decrease in income, and *n* = 401 (16.5%) reported an increase. The main reported reasons for a decreased income were unemployment/job loss/end of the contract (*n* = 192, 30.5%), perceived decreased purchasing power due to inflation (*n* = 155, 24.6%), having businesses affected by the pandemic (*n* = 96, 15.3%), and the end of the scholarship/social benefits (*n* = 38, 6.0%). For an increased income, reported reasons were getting a new job/changing workplace (*n* = 152, 40.6%), getting a paid internship (*n* = 67, 17.9%), getting a scholarship to take a graduate course (master, doctor or postdoc; *n* = 44, 11.8%), and getting a promotion (*n* = 34, 9.1%)–multiple answers were possible.

The three groups of students with different self-rated income changes during the pandemic (no change, decreased income, increased income) showed the following PHQ-9 scores: no change: *M* = 11.97, *SD* = 7.20; decreased income: *M* = 15.19, *SD* = 7.35; and increased income: *M* = 11.83, *SD* = 7.28. The level of depressive symptoms differed significantly between the groups, Welch’s *F*(2,999.499) = 48.450, *p* < 0.001, η^2^ = .04. Tukey HSD post-hoc-analysis revealed that the significant main effect was based on higher PHQ-9 sum scores in the group of students reporting a decreased income than those reporting an increased income or reporting no income change (both *p* < .001). There was no significant difference between the latter two groups (*p* = .934).

One-way ANOVAs were conducted to compare the level of depressive symptoms between the students’ income categories both before and after the pandemic. The level of depressive symptoms differed significantly between the income categories both before, Welch’s *F*(3,1222.826) = 22.827, *p* < 0.001, η^2^ = 0.03, and after the pandemic, Welch’s *F*(3,1111.541) = 29.870, *p* < 0.001, η^2^ = 0.04.

The students with different self-rated income ranges before the pandemic showed the following PHQ-9 scores: no income: *M* = 13.15, *SD* = 7.09; R$ 0-1100/mo: *M* = 14.39, *SD* = 7.22; R$ 1101-3300/mo: *M* = 12.80, *SD* = 7.38; > R$ 3301/mo: *M* = 10.64, *SD* = 7.42. The students with different self-rated income ranges after the pandemic showed the following PHQ-9 scores: no income: *M* = 13.58, *SD* = 7.15; R$ 0-1100/mo: *M* = 14.25, *SD* = 7.29; R$ 1101-3300/mo: *M* = 13.04, *SD* = 7.35; > R$ 3301/mo: *M* = 10.29, *SD* = 7.19. Post-hoc Tukey’s HSD test referring to the income before the pandemic revealed that the PHQ-9 sum score of the students that reported monthly earning more than R$ 3301 *per person* was the only one that differed significantly from all other three income categories (*p* < .001), showing the lowest levels of depressive symptoms. In addition, the R$ 0–1100 income range differed from R$ 1101–3300 (*p* < .001)–students earning the latter income range had the highest sum score in PHQ-9. There was no significant difference between the other income ranges after Bonferroni correction (all *p* > .013).

Moreover, the post-hoc Tukey HSD test referring to self-reported income after the pandemic revealed that the PHQ-9 sum score of the students that reported earning more than R$ 3301 *per person* was the only one that differed significantly from all three other income ranges (*p* < 0.001), showing the lowest levels of depressive symptoms. Furthermore, the R$ 0–1100 income range differed from R$ 1101–3300 (*p* = .010)–students earning the latter income range had the highest score in PHQ-9. There was no significant difference between the other income ranges after the pandemic after Bonferroni correction.

A one-way ANOVA was conducted to compare students of the four course levels of higher education (undergraduate/bachelor, specialization/MBA/medical residency, master, and doctor) regarding their levels of depressive symptoms (PHQ-9). The mean PHQ-9 scores are displayed in Table 2. The level of depressive symptoms differed significantly between the groups, Welch’s *F*(3,107.521) = 17.514, *p* < .001, η^2^ = 0.02, with Tukey HSD post-hoc test revealing significantly higher PHQ-9 scores in undergraduate/bachelor students in relation to all other groups as following: specialization/MBA/medical residency (*p* = .001), master (*p* = .006), doctor (*p* < .001). There was no significant difference between the students enrolled in specialization/MBA/medical residency, master, and doctor degrees after Bonferroni correction (all *p* > .013).

**Table 2:** Descriptive and statistical analysis of the mental health measures regarding the course levels students are currently enrolled in (*n* = 2,437).

Mental health measures	Total sample	Bachelor^a^	*Lato sensu* ^b^	Master	Doctor	Test value
Depressive Symptoms (PHQ-9) (*M*, *SD*)	12.85 (7.40)	13.6 (7.37)^i^	7.91 (5.37)^ii^	12.33 (7.33)^ii^	11.5 (7.32)^ii^	Welch’s *F*(3,107.521) = 17.514, *p* < .001, η^2^ = 0.02
Perceived Stress^c^ (PSS-4) (*M*, *SD*)	7.81 (2.52)	8 (2.50)^i^	6.06 (2.21)^ii^	7.74 (2.52)^i,iii^	7.49 (2.5)^ii,iii^	Welch’s *F*(3,77.571) = 7,410, *p* < .001, η^2^ = 0.01
Loneliness (UCLA-3) (*M*, *SD*)	5.25 (2.35)	5.64 (2.30)^i^	4.39 (1.55)^i,ii^	4.81 (2.33)^ii^	4.66 (2.30)^ii^	Welch’s *F*(3,108.097) = 32,182, *p* < .001, η^2^ = 0.04
Social Support (ESSI) (*M*, *SD*)	18.04 (5.15)	17.31 (5.29)^i^	19.87 (3.15)^i,ii^	18.94 (4.81)^ii^	19.08 (4.81)^ii^	Welch’s *F*(3,109.110) = 24,891, *p* < .001, η^2^ = 0.03
Self-efficacy (GSE) (*M*, *SD*)	28.23 (5.92)	27.35 (5.98)^i^	31.35 (3.60)^ii,iii^	28.66 (5.87)^ii^	29.93 (5.41)^iii^	Welch’s *F*(3,109.065) = 33,583, *p* < .001, η^2^ = 0.04
Resilience^c^ (BRS) (*M*, *SD*)	2.75 (0.86)	2.67 (0.85)^i^	3.28 (0.80)^ii^	2.84 (0.87)^ii^	2.88 (0.88)^ii^	Welch’s *F*(3,101.367) = 11,706, *p* < .001, η^2^ = 0.01

PHQ-9: Patient Health Questionnaire-9; PSS-4: Perceived Stress Scale; ESSI: ENRICHED Social Support Inventory; UCLA-3: UCLA 3-Item Loneliness Scale; GSE: General Self-Efficacy Scale; BRS: Brief Resilience Scale.

The different superscript letters ^i,ii,iii^ indicate the differences between the groups.

^a^ Bachelor and undergraduate students were considered.

^b^ *Lato sensu* refers to all specialization/MBA/Medical residency students.

^c^ Reduced sample due to missing data (PSS-4, *n* = 1,708; BRS, *n* = 2,393).

Regarding suicidality, a chi-square test showed there was a statistical difference in the number of students who answered PHQ-9 item 9 ≥ 1 (“thoughts that you would be better off dead, or of hurting yourself”), χ^2^(3) = 36.590, *p* < .001, when comparing the four groups of students: undergraduate/bachelor (66.1%, *n* = 534), specialization/MBA/medical residency (*n* = 4, 0.5%), master (*n* = 138, 17.1%), and doctor (*n* = 132, 16.3%).

A one-way ANOVA was conducted to compare students of the four course levels of higher education (undergraduate/bachelor, specialization/MBA/medical residency, master, and doctor) regarding perceived stress, loneliness, social support, self-efficacy, and resilience–the mean scores of these scales and the statistical analysis are displayed in Table 2. The level of perceived stress, loneliness, social support, self-efficacy, and resilience differed significantly between the groups of students. Overall, post-hoc-analysis revealed that undergraduate/bachelor students presented the highest levels of perceived stress and loneliness and the lowest levels of social support, self-efficacy, and resilience.

### Mental health measures

The mean PHQ-9 score of the total sample was 12.85 (*SD* = 7.40), while *n* = 1,488 (61.1%) participants reported a sum score of 10 or more, indicating clinically relevant depressive symptoms, and *n* = 808 (33.2%) reported suicidal thoughts on at least several days per week over the past two weeks. Descriptive statistics (means and standard deviations) of perceived stress, social support, loneliness, self-efficacy, and resilience are displayed in Table 2.

Regarding mental disorders, *n* = 887 (36.4%) of students reported to have been diagnosed with the following disorders: anxiety disorder (*n* = 568, 23.3%), unipolar depression (*n* = 433, 17.8%), attention deficit hyperactivity disorder (*n* = 118, 4.8%), bipolar disorder (*n* = 108, 4.4%), obsessive-compulsive disorder (*n* = 67, 2.7%), personality disorder (*n* = 49, 2.0%), eating disorder (*n* = 92, 3.8%), and others (*n* = 157, 6.4%); multiple answers possible. Also, *n* = 316 (35.6%) participants indicated they were currently not receiving any treatment for mental disorders, whereas *n* = 131 (14.8%) were in psychotherapeutic treatment, *n* = 195 (22.0%) were taking medication, and *n* = 238 (26.8%) reported both taking medication and receiving psychotherapeutic treatment. Among those that reported not having been diagnosed with a mental disorder in the past, *n* = 202 (13.1%) indicated they were currently receiving psychotherapy, *n* = 51 (3.3%) reported they were taking medication, and *n* = 43 (2.8%) reported taking both medication and receiving psychotherapy.

Regarding alcohol consumption, from *n* = 2,421 participants whose data were available, *n* = 578 (23.7%) participants consumed more alcohol during the pandemic, *n* = 1,241 (50.9%) reported no change, and *n* = 600 (24.6%) reported they consumed less alcohol. Regarding drug consumption, *n* = 229 (9.4%) reported using more drugs, *n* = 2,053 (84.2%) indicated no change, and *n* = 138 (5.7%) reported consuming less drugs during the pandemic. The mean score of the AUDIT-C hazardous alcohol use subscale was *M* = 2.8 (*SD* = 2.52); the frequency of drinking alcohol is displayed in Table 3.

**Table 3:** Alcohol and drug consumption and changes during the COVID-19 pandemic based on AUDIT-C (*n* = 2,421).

Variable	*n*, %
Current alcohol consumption	
Abstinent	547 (22.6)
Less than once a month	729 (30.1)
2–4 times a month	768 (31.7)
2–3 times a week	301 (12.4)
4 or more times a week	76 (3.1)
Change in drinking behavior during the COVID-pandemic^a^	
Less	600 (24.8)
No change	1,241 (51.3)
More	578 (23.9)
Current drug or substance use	
Abstinent	2,074 (85.7)
Less than once a month	169 (7.0)
2–4 times a month	57 (2.4)
2–3 times a week	28 (1.2)
4 or more times a week	93 (3.8)
Change in drug/substance use during the COVID-pandemic^a^	
Less	138 (5.7)
No change	2,053 (84.8)
More	229 (9.5)

AUDIT-C: subscale of the Alcohol Use Disorders Identification Test.

^a^ Reduced sample due to missing data.

Concerning weight changes in the last three months, *n* = 741 (30.6%) reported no weight change, and *n* = 1,681 (69.4%) indicated their weight changed–among the latter, *n* = 1,161 (69.1%) reported having gained weight, while *n* = 520 (30.9%) reported having lost weight, and *n* = 827 (73.0%) connected their weight changes to the pandemic.

To examine predictors of depressive symptoms, an explorative multiple regression analysis was conducted with the available data of *n* = 2,394 (see Table 4). The variables included by enter-method in the model explained a significant amount of variance in the level of depressive symptoms, *F*(16,2377) = 114.455, *p* < .001, *R*^2^ = .43 (adjusted *R*^2^ = .43).

**Table 4:** Linear regression analysis for predictors of depressive symptoms (PHQ-9) (*n* = 2,394).

Variable	Unstandardized *B*	SE *B*	Standardized *B*	95% Confidence Interval (CI)	*t*	*p*	
Age	-.023	.018	-.026	-0.058,0.013	-1.257	.209	
Gender							
Female	.163	.0249	.011	-0.325,0.651	0.654	.513	
Diverse	1.207	.962	.020	-0.680,3.095	1.255	.210	
Being parent	0.852	.431	.039	0.008,1.697	1.979	.048	
Residential status	-0.040	.353	-.002	-0.733,0.653	-0.113	.910	
Relationship status	-0.647	.257	-.044	-1.150,-0.144	-2.523	**.012**	
Migration background	0.029	.775	.001	-1.491,1.549	0.038	.970	
Decreased income	1.528	.263	.093	1.013,2.043	5.820	**< .001**	
Psychiatric conditions prior to pandemic	1.365	.251	.089	0.872,1.858	5.428	**< .001**	
Chronic somatic conditions	0.913	.315	.045	0.295,1.532	2.896	**.004**	
Positive aspects of the pandemic	1.112	.242	.074	0.639,1.586	4.604	**< .001**	
Negative aspects of the pandemic	-0.782	.612	-.020	-1.982,0.417	-1.279	.201	
Self-efficacy (GSE)	-0.252	.025	-.202	-0.300,-0.204	-10.245	**< .001**	
Social support (ESSI)	-0.114	.029	-.079	-0.171,-0.057	-3.911	**< .001**	
Loneliness (UCLA-3)	0.873	.068	.278	0,740,1.006	12.866	**< .001**	
Resilience (BRS)	-1.476	.170	-.173	-1.809,-1.142	-8.682	**< .001**	
*R*^2^ (*R*^2^ adjusted)	.435 (.431)						
*F*	114.455						
*p*	**< .001**						

Gender variable recoded into dummy variables "female" and "diverse". Relationship status dichotomised into "in a relationship" and "single". Being parent dichotomised into "being parent" and "not being parent". Bold font indicates statistical significance, *p* < .05.

GSE: General Self-Efficacy Scale; ESSI: ENRICHED Social Support Inventory; UCLA-3: UCLA-3 Item Loneliness Scale; BRS: Brief Resilience Scale.

Being single (*p* = .012), having the income decreased during the pandemic (*p* < .001), having a previous mental illness (*p* < .001), having a somatic condition (*p* = .004), not finding positive aspects in the pandemic (*p* < .001), lower self-efficacy (*p* < .001), lower social support (*p* < .001), lower resilience (*p* < .001), and higher experienced loneliness (*p* < .001) were significantly related to higher levels of depression. Age, being female, being diverse, living alone, having a migration background, and finding negative aspects of the pandemic did not show any significant association with depressive symptoms (all *p* > .05).

### COVID-19 pandemic: Attitudes towards social distancing and vaccination, and perceived burdens

Overall, 96.1% (*n* = 2,343) of students indicated they experienced negative aspects, while 60.3% (*n* = 1,471) reported they experienced positive aspects during the pandemic. Also, 86.0% (*n* = 2,095) of participants indicated they were rather or fully worried about COVID-19, and 61.0% (*n* = 1,486) reported feeling personally rather or fully in danger because of COVID-19. Most participants (*n* = 2,138, 87.7%) agreed or fully agreed with the statement that the social distancing/isolation measures hit the students hard, and 76.9% (*n* = 1,874) rather or fully agreed with the statement that the pandemic completely affected their academic activities.

Most participants (*n* = 2,295, 94.2%) supported or fully supported the use of masks and the personal and environmental sanitary measures to slow down the spread of the coronavirus. Accordingly, 91.8% (*n* = 2,238) rather or fully disagreed that the social distancing measures, the use of masks, and the personal and environmental sanitary measures caused more harm than benefits.

Most of the students disagreed or highly disagreed with the statements that the widespread fear about the coronavirus was exaggerated (*n* = 1,879, 77.1%) and that the pandemic was part of a larger conspiracy (*n* = 2,191, 89.9%). Further, *n* = 1,985 (81.5%) rather or highly disagreed with the statement that they felt responsible for the COVID-19 crisis.

When asked about stockpiling, *n* = 508 (20.8%) reported stockpiling behavior, especially regarding food, hygiene products, and medicine.

Regarding COVID-19 infection, *n* = 631 (25.9%) participants reported they had already been infected, and *n* = 480 (76.1%) reported their infection was proved by a test. Also, *n* = 1,732 (71.0%) had someone in their household or family member infected, 67.7% (*n* = 1,650) had a friend or a neighbor, and 57.4% (*n* = 1,398) had an acquaintance who was infected with the coronavirus. Only 1.0% (*n* = 24) reported not knowing anyone that was infected with the coronavirus. Further, *n* = 1,957 (80.3%) participants reported knowing someone who died because of COVID-19.

The participants were retrospectively asked about having medical or psychological appointments during the months of March to June of 2021, when the numbers of deaths were highest in Brazil. Face-to-face appointments were the most common way participants reported they used when having a medical appointment (*n* = 1,166, 47.8%), followed by those who used a videoconference (*n* = 433, 17.8%). The videoconference was the main communication means used by the students when they had psychological appointments (*n* = 735, 30.2%). Some participants indicated that despite being in need of help, they did not get medical (*n* = 102, 4.2%) or psychological (*n* = 196, 8.0%) appointments.

The students were retrospectively asked about having direct and indirect personal contact during the months of March to June 2021: *n* = 1,230 (50.5%) participants reported having no personal contact with other people outside their household during that period, *n* = 834 (34.2%) reported having had contact once or twice a week, and *n* = 374 (15.4%) three or more times per week; *n* = 1,142 (46.9%) reported having indirect contact (e.g., phone or video call) every day or several times a day.

Regarding vaccination against COVID-19, 97.3% (*n* = 2,372) of the participants reported to be fully vaccinated, and 1.6% (*n* = 40) partly vaccinated. Concerning their attitudes towards vaccines in general, 96.9% (*n* = 2,361) reported being favourable or rather favourable, 1.1% (*n* = 26) partly, 1.3% (*n* = 32) rather or hostile refusing, and 0.8% (*n* = 18) preferred not to say or did not know.

The sources to get information about the COVID-19 pandemic most commonly reported by the participants were online newspapers (*n* = 1,544, 63.3%), official websites (*n* = 1,366, 56.1%), television (*n* = 1,150, 47.2%), Instagram (*n* = 899, 36.9%), Twitter (*n* = 560, 23.0%), and Facebook (*n* = 368, 15.1%); multiple answers were possible.

When asked how their lifestyle was affected by the pandemic-related restrictions, *n* = 518 (21.3%) participants agreed or fully agreed with the statement that they felt severely restricted by the social distancing measures. Despite the finding that *n* = 1,153 (47.3%) rather or fully agreed with the statement that "overall, it is good to me not to go out so much and have less contact with other people," *n* = 1,541 (63.2%) rated social life as an aspect that was significantly restricted. Cultural activities and exercising were also rated as significantly restricted by *n* = 1,420 (58.3%) and *n* = 887 (36.4%), respectively.

Hobbies were rated as being significantly restricted by *n* = 737 (30.2%) participants, while *n* = 509 (20.9%) reported it as not being restricted. Dating was reported as significantly restricted by *n* = 692 (28.4%) participants, while being not restricted by *n* = 536 (22.0%). Sexual activity was reported to have been significantly restricted by *n* = 678 (27.8%), while it was not restricted to *n* = 694 (28.5%) participants. Healthy eating was pointed out as an aspect that was not affected by *n* = 996 (40.9%) participants. Alcohol and drugs were reported to be significantly restricted by *n* = 272 (11.2%) participants, while *n* = 643 (26.4%) reported it was not restricted.

## Discussion

This survey presents remarkable and, to our knowledge, the first comprehensive information regarding Brazilian students’ mental health, attitudes, social and emotional aspects, and some burdens of the COVID-19 pandemic. The survey was conducted when at least half of the Brazilian population was fully vaccinated, and the rates of contagious and deaths were considerably lower when compared with the months when the rates of new deaths per million reached its highest point in Brazil. Nevertheless, as already pointed before [1, 2, 4, 8, 10], the results of this survey also show that the students are facing the burdens of the pandemic substantially and that the psychosocial and emotional impact of the pandemic may be long-lasting and extensive.

There were differences between drop-out and completers. Completers were more often parents (13.7% in this sample vs. 10.5% in drop-out), were enrolled in higher course levels (20.7% enrolled in doctor course in the sample vs. 13.0% in drop-out), and had a higher income range before and after the pandemic–especially in the category income higher than R$ 3301. Drop-out students were more often enrolled in lower course levels (66.7% of drop-outs were enrolled in undergraduate/bachelor courses vs. 57.6% in this sample) and had lower income ranges before and after the pandemic, especially in the income range of R$ 0–1100. The number of students who did not want to report their income after the pandemic was higher among participants who dropped out (5.4%) than in the completers’ sample (3.0%).

Similarly to previous studies, most participants were female (64.8%), similar to previous studies [40, 41, 69]. Moreover, most university students (both undergraduate/bachelor and graduate students) in Brazil are female [70], which is representative of Brazilian university students.

### Mental health measures

The level of depressive symptoms in this sample was higher than in the general population in Brazil [71–73], although, in the study of Goularte and colleagues, depression was present in 68% of the sample [5]. Nevertheless, this result was very similar to other study conducted from September to October 2020 with *N* = 1,224 students from five public universities also in Parana state [51].

The mean PHQ-9 score of 12.85 in this sample is considered a status of moderate depressive symptoms (PHQ-9 score between 10 and 14), and a score higher than 10 represents clinically relevant depressive symptoms [56]. Besides, every third student reported suicidal thoughts on at least several days per week over the past two weeks.

Levels of depressive symptoms differed between participants with different self-rated income changes during the pandemic, with higher PHQ-9 sum scores in the group of students reporting a decreased income than those reporting an increased income, which was similarly pointed by Lopes and Nihei [51]. Accordingly, students with higher income both before and after the pandemic presented lower PHQ-9 sum score.

Although the financial situation did not change for 55.5% of the students, the consequences might be critical for those whose income decreased due to the pandemic. Post-hoc-analysis revealed a significant difference in depressive symptoms in this sample between students with decreased income in relation to those whose income stayed stable or had their income increased. Job loss/unemployment and inflation were pointed as the main reasons the students had an income decrease, which might illustrate the burdens of the pandemic in the Brazilian economy.

Furthermore, the level of depressive symptoms differed significantly between the income categories before and after the pandemic, in which students with higher income ranges (more than R$ 3301 *per person)* showed lower levels of depression. Also, students earning R$ 0–1100 presented higher levels of depression in relation to those earning R$ 1101–3300. Low income and lower level of education were also reported to be associated with higher severity of symptoms in the general Brazilian population by Goularte and colleagues [5]. It highlights the importance of having financial support programs for students and the need to update and correct the scholarship grants according to the inflation rate [69].

Moreover, regarding differences between the course levels, post-hoc-analyses revealed that undergraduate/bachelor students scored higher for depressive symptoms than the students enrolled in specialization/MBA/medical residency, master, and doctorate courses. These results should be carefully observed as students seem to have an increased risk of developing mental health problems since psychiatric disorders often first appear between the ages of 18 and 24 [74]. It reveals that younger adults are experiencing more depressive symptoms, which indicates that further studies are needed to investigate mental health outcomes and special prevention programs–therefore, longitudinal studies or comparison studies between first-year students and seniors could be particularly useful. It also indicates that there might be differences in characteristics in these populations that require different approaches to mental health promotion and prevention interventions in a university environment, based on these populations’ specificities in psychological distress, as already suggested [6].

Further, as a cultural practice, it is notable that the number of students who reported being currently in psychotherapeutic treatment, taking medication, or both, despite not having a diagnosis. Anxiety and depression were the most common mental disorders reported by the participants, which is comparable with the data of the general population in Brazil [5].

Regarding alcohol and drug consumption, the results show that most participants reported they consumed equal or fewer amounts, which could be related to fewer social gatherings (e.g., parties or events) due to social distancing and the need to prioritize their expenses, for example.

As expected, the regression analyses showed that having lower self-efficacy, lower social support, lower resilience, and higher experienced loneliness were related to higher levels of depression. Regarding health conditions, having a previous mental illness or a chronic somatic condition were risk factors for depressive levels, which is corroborated by previous studies [10, 72]. In addition, being in a relationship (vs. being alone) seems to be a protective factor, while not finding positive aspects in the pandemic revealed to be a predictor for depression in the model [10]. Moreover, as the financial aspect already pointed out earlier [8, 10, 72], having income decrease during the pandemic was a predictor to depressive symptoms.

Surprisingly, differently from what was observed in other studies with university students and the general population [5, 21, 51, 73, 75], this sample did not show a significant statistical difference among female or diverse genders as predictor variables of depressive symptoms, and neither to not being a parent [5, 8, 21, 27, 73].

### COVID-19 pandemic: Attitudes towards social distancing and vaccination, and perceived burdens

Despite the fact that when the survey was conducted, 97.3% of the students reported being fully vaccinated or partly vaccinated (1.6%) against COVID-19, most of them (86.0%) indicated they were rather or fully worried about COVID-19 and felt personally in danger (61.0%). Despite the late start of the vaccination in Brazil–similar to other low-income countries [43, 44] –the country’s solid immunization strategy within the universal healthcare system and settled infrastructure might have contributed to the high vaccination acceptance among the participants [48]. Also, higher income and educational level are some of the factors related to higher willingness to vaccinate [76]. Different studies have indicated that higher levels of trust in information from government sources [77], knowledge about the COVID-19 vaccine and risk perception of COVID-19, and supporting attitude towards vaccinations in general [78] are related to vaccine acceptance [79]. Considering the kind of sources to get information about the COVID-19 pandemic most reported by the participants, it could be argued that trustworthy sources of information may have positively affected how students behaviorally [80] and emotionally [23] responded to mitigate the spread of the coronavirus.

Although Brazil did not have homogenous governmental measures to slow down the spread of the coronavirus, and this was sometimes controversial [81–83], students supported the restrictions and health measures prescribed by health professionals. In this regard, studies have shown a connection between compliance to protective behaviors, such as mask-wearing, and individual characteristics and sociodemographic factors [84]. Being female, having a higher age, having a higher income, living in urban areas, and having a university degree were shown to be related to a higher likelihood of wearing a mask [84–86]. In terms of beliefs and attitudes, the perceived severity of COVID-19 and perceived benefits have been also associated with the likelihood of wearing a mask [87]. Self-efficacy has been associated with vaccine acceptance and mask-wearing [87, 88].

Even if it is widely known that Brazil became one of the countries with the highest tolls of deaths due to COVID-19, the high number of students who knew someone who died because of COVID-19 is still surprising. It indicates that grief, as a collective shared experience, became an important aspect to be considered when it comes to students’ mental health. Further, students presented elevated levels of perceived stress and loneliness, low levels of social support, self-efficacy, and resilience. These results are comparable to other international studies with university students [8, 10].

Due to social distancing measures, social life and cultural activity were reported to have been significantly restricted during the pandemic by the students. If social support was considered a strong protective factor against mental health problems and has been suggested as a valid coping strategy in this pandemic [11], the decrease in its availability under pandemic conditions might have had the opposite impact. Accordingly, Chung and colleagues [89] suggested that higher compliance to protective behaviors (e.g., regular handwashing, regular ventilation maintenance, and social distancing) seems to be related to less psychosocial distress, whereas reduced gatherings with family or friends were associated with higher psychological distress.

Moreover, the overload of information and misinformation regarding the pandemic, the uncertainty regarding their academic and professional future, the socioeconomic long-lasting side effects of the pandemic, and grief–to mention a few burdens–might lead to decreased quality of life [83] and intermediate socioemotional aspects.

## Strengths and limitations

This study has some limitations. First, because the data collection was online, the sample comprised only students with internet access. Second, in the multiple linear regression, the perceived stress score could not be inserted in the model due to missing data, which probably improved its variance. Third, because Brazil has a multidimensional inequality and presents a very diverse income configuration [90], the results on income and income changes during the pandemic should not be generalized to the population of students in Brazil. It reflects a particular group of higher education students from south Brazil. However, this study also has several strengths. It has a relatively large sample compared to other studies with the same population in Brazil. Besides, it uses several statistical tests to explore the relationships examined here, mainly regarding the differences among students from different degree levels, which has been a topic neglected so far.

## Conclusion

This study showed high levels of depressive symptoms and suicidal ideation among students of the Federal University of Parana, which places them as a vulnerable group for mental disorders. Being single, having income decreased during the pandemic, having a previous mental illness, not finding positive aspects in the pandemic, having lower self-efficacy, lower social support, lower resilience, and higher experienced loneliness were risk factors for higher depressive symptoms. The results suggest that healthcare providers and universities must recognize and address mental health issues. Also, social policies must be enhanced, especially in a developing country such as Brazil. Psychosocial preventive and supportive programs can be helpful [91], either face-to-face or online, especially considering the long-lasting and extensive effects of the pandemic on students’ mental health and well-being [10].
